# Parallel-SymD: A Parallel Approach to Detect Internal Symmetry in Protein Domains

**DOI:** 10.1155/2016/4628592

**Published:** 2016-09-26

**Authors:** Ashwani Jha, K. M. Flurchick, Marwan Bikdash, Dukka B. KC

**Affiliations:** Department of Computational Science and Engineering, North Carolina A&T State University, Greensboro, NC 27411, USA

## Abstract

Internally symmetric proteins are proteins that have a symmetrical structure in their monomeric single-chain form. Around 10–15% of the protein domains can be regarded as having some sort of internal symmetry. In this regard, we previously published SymD (symmetry detection), an algorithm that determines whether a given protein structure has internal symmetry by attempting to align the protein to its own copy after the copy is circularly permuted by all possible numbers of residues. SymD has proven to be a useful algorithm to detect symmetry. In this paper, we present a new parallelized algorithm called Parallel-SymD for detecting symmetry of proteins on clusters of computers. The achieved speedup of the new Parallel-SymD algorithm scales well with the number of computing processors. Scaling is better for proteins with a larger number of residues. For a protein of 509 residues, a speedup of 63 was achieved on a parallel system with 100 processors.

## 1. Introduction

**Figure 1:**
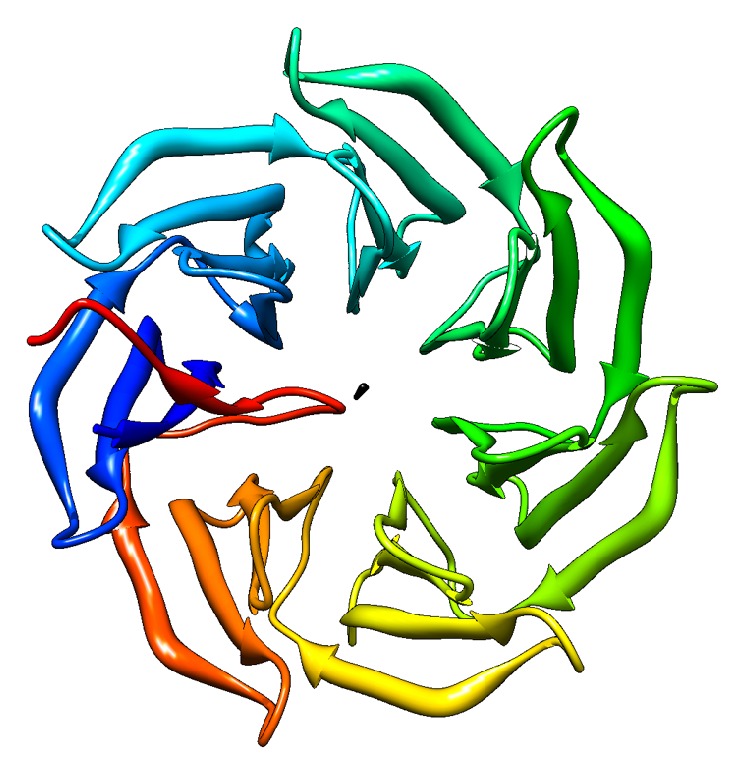
Symmetric 7-bladed beta propeller. The black dot shows the axis of rotation.

Not only multimeric proteins and protein complexes, but also the repeating units in monomeric proteins are arranged in a symmetric manner. We previously reported a method called SymD [1] and a webserver [2] based on SymD to determine internally symmetric proteins. Using SymD [1], around 10% of SCOP 1.73 ASTRAL40 domain database [3] is determined to be internally symmetric.  Figure 1 shows examples of a symmetric 7-bladed beta propeller.

In comparison to other existing algorithms, SymD has proven to be a robust algorithm to determine internal symmetry in proteins. On the other hand, the exponential increase in computer power has made it possible to perform complex matrix operations in much less time. Many techniques exist for detecting the internal symmetry in protein domains. Some techniques use the structure alignment program [4–8] and others use periodic occurrence of repeats along the primary sequence [9–12].

The SymD algorithm makes use of an alignment scan procedure where the original protein structure is aligned to copies of itself obtained by circular permutation of all possible numbers of residues. A webserver of SymD algorithm has also been made publicly available [2]. SymD has demonstrated its ability to find a large number of symmetric proteins across various protein folds. SymD performs quite well in terms of accuracy when compared to other protein symmetry detection algorithms. Some algorithms detect a specific domain within the protein structure really well, but when tested extensively with all domains they tend to perform poorly. But SymD performs remarkably well for all protein domains and particularly for beta propellers where it detects the protein symmetry with 100 percent accuracy. Despite its robustness, emerging problems in bioinformatics require dramatically faster methods of detecting internal symmetry in protein domains, and hence SymD requires improvement.

The large size of protein structures presents a challenge to commonly used symmetry detection algorithms. The time taken to compute the symmetry of protein structure increases significantly with the size of protein structure due to the huge number of matrix operations. In spite of being consistently accurate across all protein domains, SymD lags behind in terms of speed and scalability. With little increase in input size, the speed decreases drastically, and, moreover, it does not have the capability to scale over multiple processors and multiple cores.

To reduce the limitation of SymD, we have come up with a new parallel algorithm that basically uses the same SymD design at its core but extends its capability to multiple processors and distributed computing systems which in turn help SymD to utilize multiple processors available to improve the speed of computation.

The need of parallelization arises because of this increased computational time with respect to the size of protein structures. Conversely, contemporary computers have typically multiple computing units (cores) [13]. Although aligning the original structure with one copy of the circularly permuted structure can be done in a much lesser time, performing the alignment scan for all possible numbers of circularly permuted structures requires high computational time. Consequently, as the size of protein structure increases, faster symmetry detection methods are required to handle the increasing computational load.

The message passing interface (MPI) standard for communication in parallel computing offers a solution to this problem [14]. The central processing units (CPUs) of modern computers have multiple cores that are separately programmable and can, when used proficiently, offer a significant increase in computation speed over single CPUs [15]. Furthermore, parallelization has been applied effectively to many problems in bioinformatics [16, 17].

In this article, we present Parallel-SymD, the parallelized SymD algorithm for detection of internal symmetry in protein domains. This new algorithm that detects symmetry in protein domains is especially suited to efficient execution on multiple CPUs and computer of varying power interconnected in a network, which are available in contemporary computing platforms or computing clouds. When detecting the symmetry of a protein that has 509 residues, Parallel-SymD is around 63 times faster compared to the existing SymD algorithm when run in a parallel system using 100 processors. We explain the SymD algorithm, provide the description of our new Parallel-SymD algorithm, and discuss the performance and comparison between the two algorithms.

## 2. Materials and Methods

We describe Parallel-SymD algorithm by describing in short the original SymD algorithm [1] followed by the detailed algorithmic description of the Parallel-SymD algorithm.

### 2.1. Overview of SymD Algorithm

The SymD [1] algorithm works by performing the alignment scan between the original structure and copies of itself, circularly permuted by all possible numbers of residues. It finds the score that determines the symmetricity of the original structure. Briefly, the algorithm first makes a copy of the original structure and circularly permutes its residues at all positions from 1 to *N* − 3, where *N* is the number of residues of the protein. The algorithm then finds the best non-self-structural alignment between the original and each of the *N* − 3 permuted structures. This process is called the “*alignment scan.*” The best alignment with each permuted structure is obtained using the RSE algorithm [18], which iterates a two-step cycle. In the first step, the Kabsch algorithm [19, 20] is used to optimally superimpose the two structures by minimizing the weighted sum of squares of the distances between aligned pairs of residues. Then, in the second step, optimal structure-based sequence alignment is obtained from the superimposed structures using the SE algorithm [21]. The two-step cycle is terminated when the procedure has converged or when a set number of cycles are finished. The final alignment reported is the one with the best score during the cycle. The *Z*-score [1] of the *T*-score, which is a weighted number of aligned residues, similar to the sum of the similarity matrices *S*_*ij*_ of Gerstein and Levitt [22, 23], is finally reported.

In this regard, SymD outputs the *N* − 3 alignment scores and also the position and orientation of the symmetry axis for each of the alignments. The information about the position and orientation of the symmetry axis is obtained from the transformation matrix. Finally, a protein is deemed to be symmetric if one of the *Z*-scores of the alignments is greater than the cut-off value (*Z*-score of 8 or 10). It has to be noted here that symmetricity is not exact and it depends on the scoring function and the cut-off value associated with the scoring function. In this regard, a comprehensive and systematic analysis of various scoring functions and a systematic determination of cut-off value are required. Furthermore, SymD can also provide information about repeating units in a symmetric protein.

### 2.2. Parallel-SymD Implementation

At the heart of the SymD algorithm is the “alignment scan” procedure that aligns the original structure with each of the *N* − 3 permuted structures and finds the best non-self-structural alignment between the original and each of the *N* − 3 permuted structures. Each of the iterations in the alignment scan procedure is* independent* and thus SymD algorithm is well suited for parallelization. Here, an iteration consists of finding the best non-self-structural alignment between the original and each of the *N* − 3 permuted structures and calculating optimal structure-based sequence alignment from the superimposed structures using the SE algorithm [21] and then calculating the similarity score.

Parallelization of the SymD algorithm should be accomplished in such a way that the algorithm should automatically be able to share the workload equally among the available number of processors. If we increase the number of processors, it should again redistribute the workload so that each processor will have the same amount of workload.

**Figure 2:**
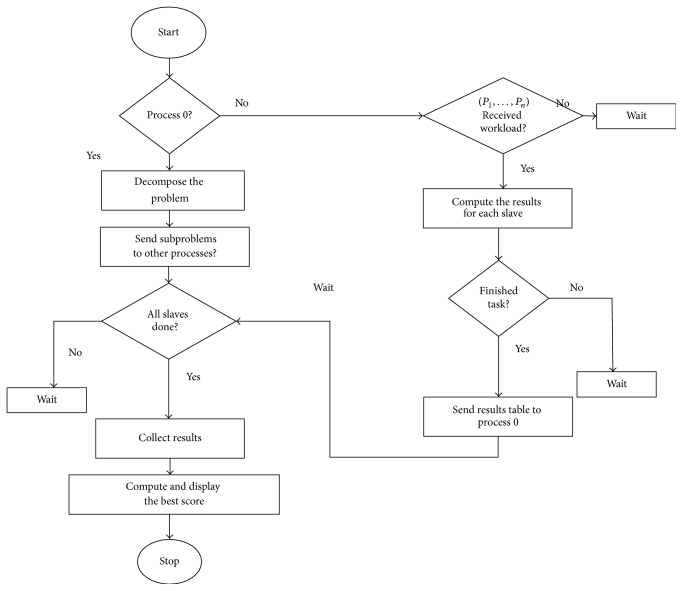
Overview of Parallel-SymD algorithm. Process 0 acts as a master node. If the rank ID of a process is not 0, then it will wait to receive the workload from process 0. After process 0 divides the problem and sends it to the rest of the processes, then each processor independently solves the subproblem and generates the results that are then sent back to the master node. Finally, the master node displays the results.

Parallelization of the time-consuming alignment scan should be accomplished in such a way that it is equally efficient in single or parallel computing systems. This implies that the parallelization methodology must incorporate automatic balancing of computation. The alignment scan between original structure and the copy of circularly permuted structure of itself is* independent* for each permutation, and thus the algorithm is* appropriate for parallelization*. Parallelization can also be achieved on the level of single computing node but such approach is not effective on parallel and distributed computing platforms with a lot of interconnected nodes. Because the alignment scan procedure implies high ratio between computation time and communication time, it can be better parallelized on a task level [15]. The Parallel-SymD algorithm is depicted in Figure 2.

A parallel platform can be represented as a set of slave nodes with a single master node. Bookkeeping is implemented as a separate process that runs on the master node. Usually, the master bookkeeping process is much simpler that the alignment scan processes and master node can run concurrently with slave nodes.

This can be achieved by removing any hard coded area that restricts the number of processors and workloads from being shared. Instead of hard coding at the compilation time, we implement the program according to the following guidelines: (a) decide at runtime how and among how many processes should the workload be distributed; (b) as soon as a new processor is available, automatically redistribute the work share among the newly available resources.

Obviously, one must check that SymD and Parallel-SymD yield the same results. To achieve this, we start by assigning the roles for each processor as a master or slave and design our program in such a way that even though the computations are done in individual processor, the results are sent back to one place and final results are shown by one processor.

For accomplishing this, we assign the role of housekeeping to the master processor (i.e., processor with rank 0). The master processor will handle the decomposition of tasks into subtasks and mapping of those subtasks to other processors, collecting the results from each processor, and analyzing those local results and generating the final answer. In this regard, an iteration of “alignment scan” is run on slave nodes. The communication between processes is implemented using a standard MPI library and consists of master node sending the “specific number of iterations of alignment scan” to the slave node that will return the best *Z*-score for the alignment scans to the master node.

As soon as the master processor gets the input and the information about the total number of processors, it reads the input protein and calculates the total number of residues in the input protein. Based on the number of processors available, the master processor then decomposes the task. If the residues lengths are not exactly divisible by the number of processes, the last processes as per the rank will be loaded with little extra work. After the decomposition of the task into subtasks, the master processor goes on a waiting phase where it seeks results from each processor. Sometimes, a process may take a long time to carry out execution and another process may complete the execution in a short time. But process 0 will not continue executing and analyzing the results until and unless it receives the result from each processor.

Once the master processor collects all the *N* − 3 alignment scores, it displays all the results. Finally, a protein is considered symmetric if the *Z*-score of the best of these alignments exceeds a certain cut-off value.

The program that activates the SymD algorithm is the same for all computing slave nodes and has two parts: first for supervising master process with process identification (ID) = 0 and second for the remaining of the slave processes. The communication between parallel processes is implemented using standard MPI library [14] and consists of a master node sending the circularly permuted structure and original structure to slave nodes that will return the symmetric scores between the two structures to the master node. Communication is short: for each alignment scan, only the matrices of the two structures are sent to slave nodes, and only the information regarding the optimal alignment is returned by each slave node to the master node. In addition, queues of permuted structures on the slave nodes serve as buffers providing slave nodes with work. Since the computation of symmetric scores involves huge matrix operations, the time lost on communication between processes is several orders of magnitude shorter than the computation time. Consequently, the restrictions posed by communication channel bandwidth and message latency are very low.

As the MPI is available in a standard form for most existing platforms [14], our approach is highly portable. The communication requirements are minimal and computational load is automatically balanced.

## 3. Results and Discussion

To validate and assess the performance of the Parallel-SymD algorithm, we performed a series of computational experiments that are presented below. The computational experiments were performed on a CRAY XC30 system. This parallel computer system is composed of 6 CPU quad-socket 8-core Intel nodes and two CPU/GPU 10-core Intel nodes with 4 K40 NVidia GPU. The operating system is Linux Kernel, CRAY CLE based on SuSE SLES release 11.3. MPICH (version 2) was used and CRAY compilers were used to compile the source program. The nodes are connected with Aries Interconnect. Please refer to [24] for the details.

### 3.1. Validation of Parallel-SymD Algorithm

**Figure 3:**
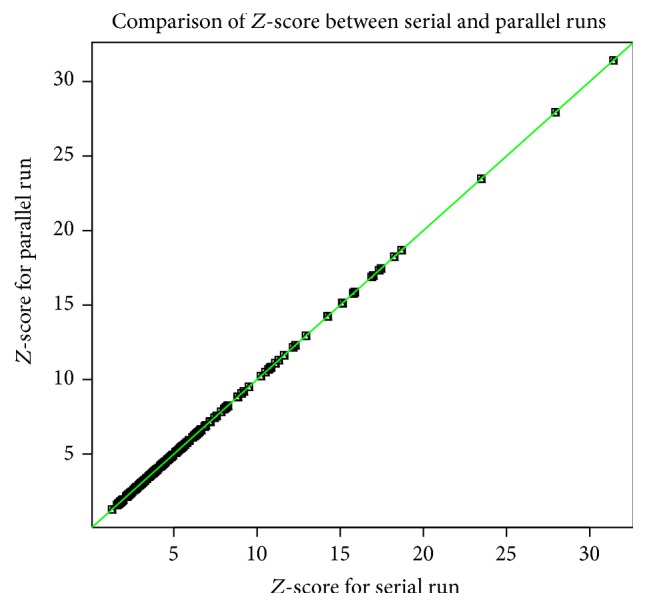
Comparison of *Z*-score between SymD and Parallel-SymD using a set of 200 proteins.

**Figure 4:**
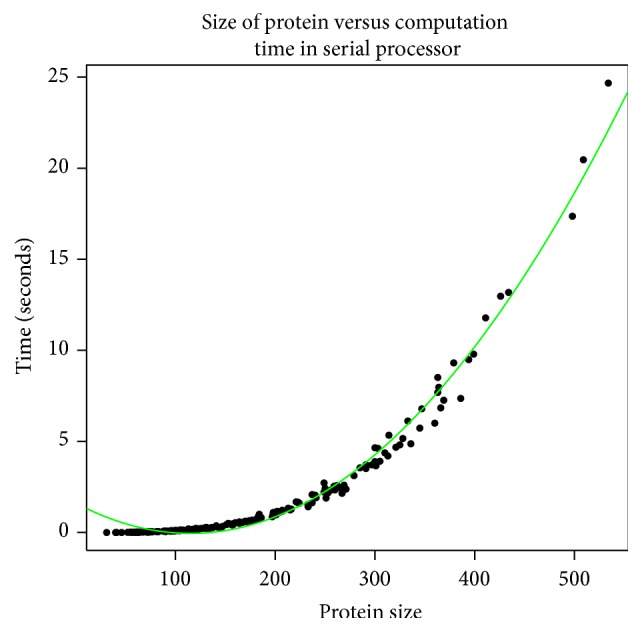
Change in computation time as the size of the protein increases for a single processor. Results obtained from 200 randomly selected proteins of various lengths on a CRAY XC30-AC parallel system using a single processor. The trend line is a quadratic fit to the data.

In order to validate that the Parallel-SymD algorithm works, we ran a set of 2000 proteins of various lengths and compared the *Z*-scores using the SymD [1] and Parallel-SymD and plotted these scores in Figure 3. It can be observed from the figure that Parallel-SymD and SymD produce the same *Z*-score for a set of 2000 proteins which validates the notion that Parallel-SymD and SymD produce the same results. We also show in Figure 4 the variation of computation time as the size of the protein increases in a single processor. Though not shown, it has to be noted that as the size of the protein increases (beyond 500 residues) the computation time increases polynomially.

### 3.2. Communication Time

Performance gain depends upon various factors like communication time (time taken for two processors to communicate, that is, sending and receiving messages), computation time (actual time taken for computation of solution), and protein size. If the protein size is increased, the communication time increases for a given processors number. For example, if we choose to use 20 processors, the communication time increases as we keep increasing the protein size.

**Figure 5:**
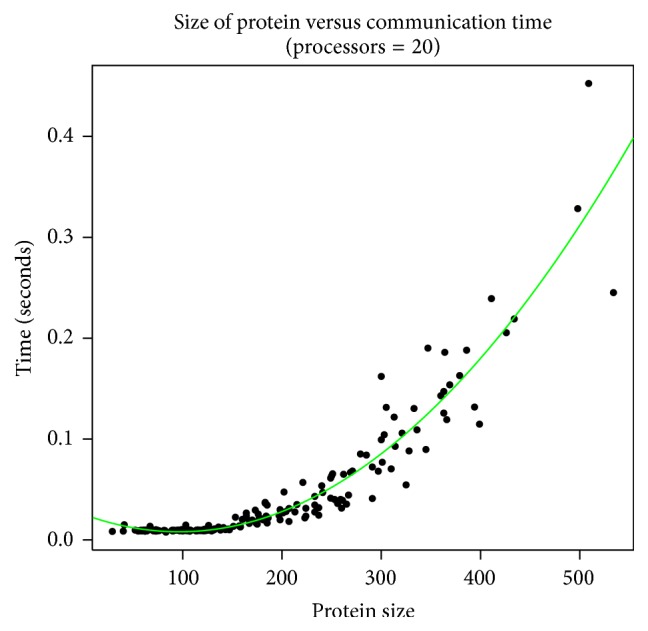
Change in communication time as the size of the protein increases for a fixed number of processors (= 20). Results obtained from 200 randomly selected proteins of various lengths on a CRAY XC30-AC parallel system using 20 processors.

A plot of communication time versus the number of residues of proteins for 20 processors is shown in Figure 5, which in general demonstrates that the communication time shows quadratic growth with increase in the number of residues.

### 3.3. Computation Time

**Figure 6:**
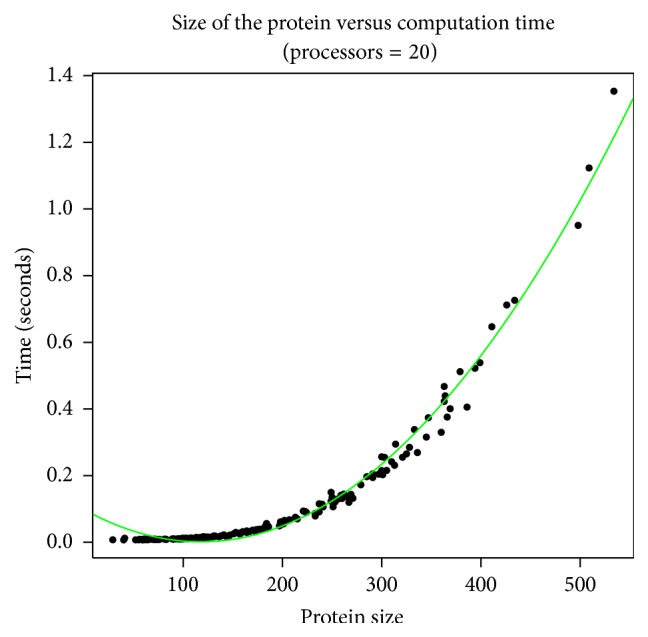
Change in computation time as the size of the protein increases for a fixed number of processors (= 20). The computation time measured is the actual time taken for computation. The results were obtained after executing 200 randomly selected proteins of various lengths on a CRAY XC30-AC parallel computer system using a constant processors number of 20.

We also examined the effects of increasing the protein size on the computation time of calculating the symmetricity of a given protein. We observed that computation time shows quadratic growth as the protein size increases when the processor count is kept constant (= 20). The results of this analysis are shown in Figure 6.

Apart from the number of residues or protein size, the communication time and computation time are also dependent on the number of processors used. If we vary the number of processors keeping the protein size constant, we could see how the communication time increases and computation time decreases as we increase the processor count.

**Figure 7:**
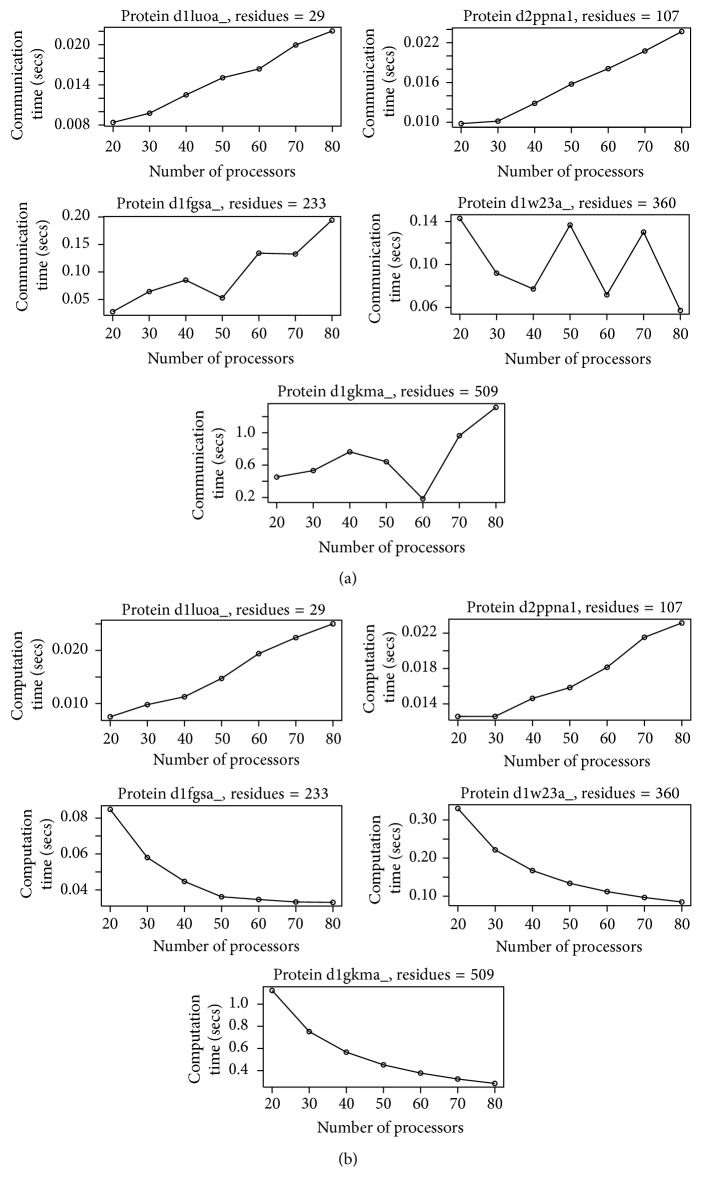
(a) Variation in communication time as we keep increasing the processors number keeping the protein size constant. This was executed on CRAY XC30-AC on five randomly selected proteins of sizes 29, 107, 233, 360, and 509. (b) Variation in computation time as the number of processors increases keeping the protein size constant as executed on CRAY XC30-AC parallel system on 5 randomly selected proteins of sizes 29, 107, 233, 360, and 509.

Figure 7(a) shows how the communication time and Figure 7(b) shows how the computation time vary as the number of processors is varied while keeping the protein size constant. We have executed this on five proteins of various lengths and each protein was run on CRAY XC30-AC parallel system using 20, 30, 40, 50, 60, 70, and 80 processors. It is interesting to note that, for smaller proteins, the computation time increases as the number of processors is increased. This is due to the fact that for smaller proteins the ratio of the cost of communication time increases compared to the computation cost. Computation time in general decreases when we increase the number of processors keeping the protein size constant.

### 3.4. Performance Gain

Next, we measured the speedup of the proposed parallelization on a number of proteins taken from ASTRAL40 dataset [3]. We have selected around 2500 proteins from the dataset to measure the impact of protein size on the performance of the Parallel-SymD algorithm.

We first ran the SymD algorithm on a single processor to find out the execution time *T*_1_ of the nonparallel version of the algorithm. Then, we ran the Parallel-SymD algorithm on 20, 30, 40, 50, 60, 70, 80, and 100 processors of a CRAY XC30-AC parallel computer system. We measured the execution time of Parallel-SymD *T*_*p*_ and calculated speedup [16] as *S* = *T*_1_/*T*_*p*_. The maximum theoretical speed also known as ideal speed is equal to (*p* − 1) since only *p* − 1 processors are involved in performing the computation.

**Figure 8:**
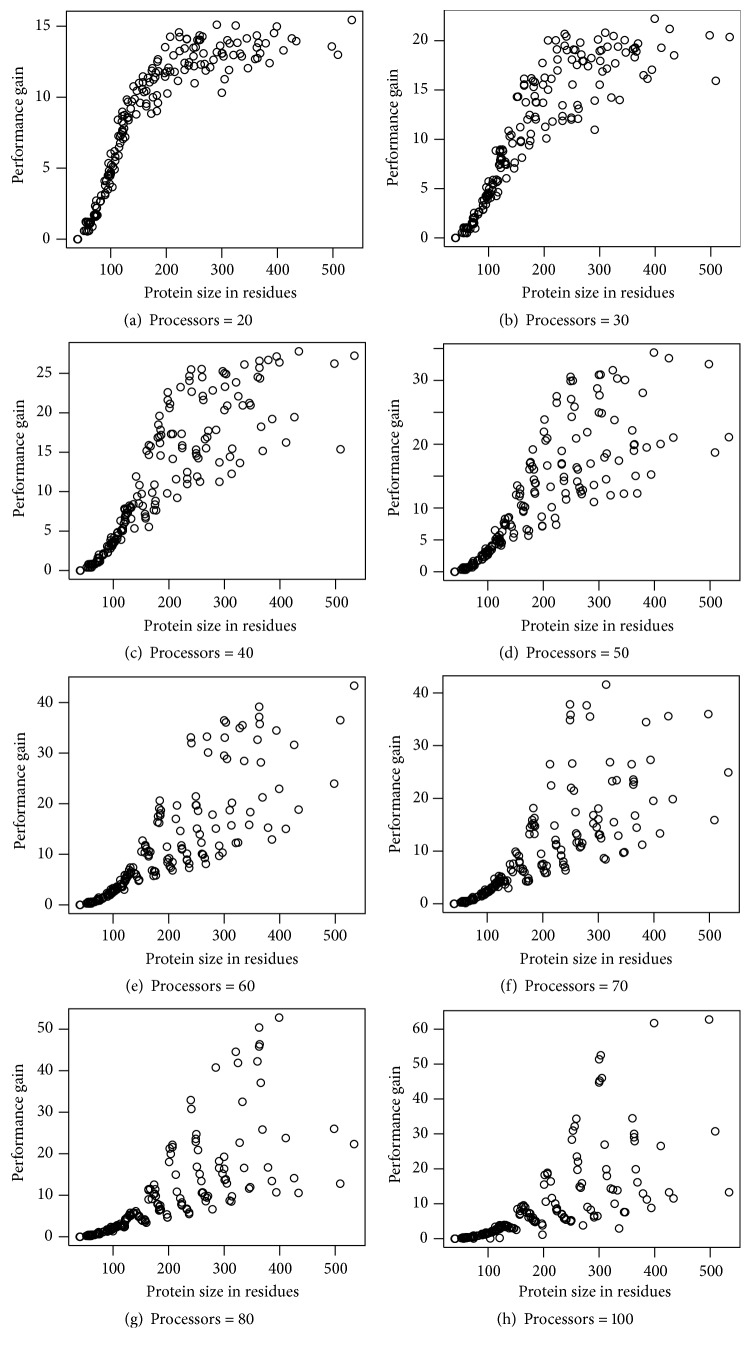
Performance gain with respect to various sizes of protein for different numbers of processors (20, 30, 40, 50, 60, 70, 80, and 100) for 200 proteins of various sizes. Even though generally the performance gain increases with the increase in the number of processors, the gain depends on the size of the protein. For larger protein, performance gain is higher than for smaller protein.

We plotted the performance gain (speedup) when Parallel-SymD is run on different numbers of processors ranging from 20 to 80. The results are shown in Figures 8(a)–8(g). It can be observed from the results that for the smaller protein of ~100 residues the speed does not increase when using 100 processors. Therefore, the smaller proteins should not be calculated on higher number of processors. The parallel scaling efficiency, which is defined as the ratio between measured speed up and ideal speedup, is in between 50% and 81%. It decreases with the number of computing nodes and increases with protein size. The speedup was close to 64 when used with 100 processors, and it was close to 55 when used with 80 processors and decreases as we decrease the number of processors. The proposed methodology is very appropriate for the network and cluster computing. Further increase of the speedup is possible by the parallelization on the level of symmetry detection algorithm itself, which will result in finer granularity of the problem and easier load-balancing of processors. In particular, we can parallelize the RSE routine that is the essential building block of the proposed symmetry detection algorithms. Future work includes exploring the efficiency of many-core and graphic processing unit (GPU) platforms in further parallelization approaches.

The advantage of the proposed Parallel-SymD is its ease of use and better performance, compared to naive parallelization. The only requirement is that the Parallel-SymD program is properly installed and that the master node is able to communicate with all slave nodes through the MPI library.

### 3.5. Postprocessing Time

**Figure 9:**
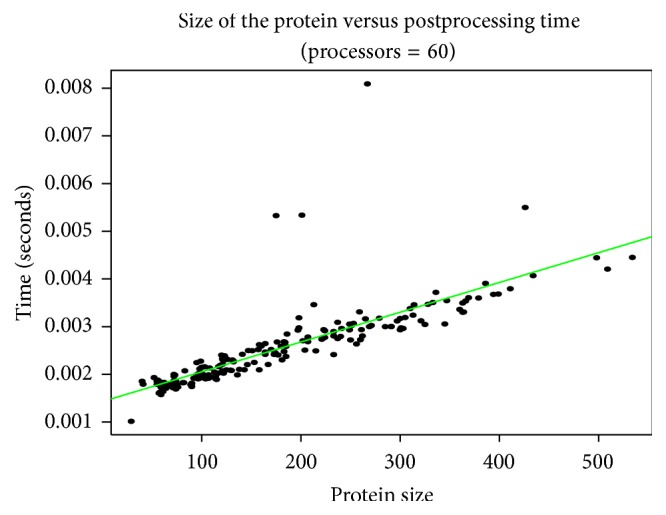
Variation in postprocessing time with increase in protein size as performed on CRAY XC30-AC parallel system using 60 processors.

We also plotted the time taken by the master node from the moment it receives data from the slave node to the point when it displays the result, postprocessing time. Since a single processor does the postprocessing, we have plotted the graph of postprocessing time against the protein size in Figure 9. It can be observed that postprocessing time is relatively insignificant compared to the computation time.

## 4. Conclusions and Discussion

In this work, we presented a parallel strategy to determine internal symmetry in a protein called Parallel-SymD. As the number of proteins structures continues to grow, it becomes very important to understand and characterize the structural features of these proteins including internal symmetry of the protein. The proposed parallel algorithm has been implemented on distributed computing system environment. The experimental results show that the algorithm presents good scalability and a nearly linear speedup. With the use of 100 processing nodes, the system achieved a 65x speedup. Thus, the proposed parallel algorithm scales well with the number of processors, enabling high performance on parallel systems.

As observed from Section 3, Parallel-SymD helped achieve a huge amount of performance gain compared with the serial version of SymD. As the performance gain depends upon various factors like computation time, communication time, size of protein, and so forth, thus the performance gain does not necessarily increase in the same ratio as the number of processors for all protein sizes.

We have also characterized speedup of the algorithm by running the algorithm using 20, 30, 40, 50, 60, 70, 80, and 100 processors. Although we were not able to achieve the ideal speedup in each case (which should be 19, 29, 39, 49, 59, 69, 79, and 99 times, resp.) (total processors − 1), however, we were able to achieve a nearly linear speedup. To be precise, in the case of 20 processors, we achieved performance gain closer to 81% of the ideal value. Similarly, in the case of 80 processors, we achieved performance gain closer to 74% of the ideal value.

Finally, Parallel-SymD approach is one of the first types of approaches to detect symmetricity in proteins which harnesses the massive parallel architecture of existing computational infrastructures. To facilitate the use of Parallel-SymD source code, an executable of the program can be obtained from the corresponding author.
